# Bioclimatic and Landscape Factors drive the Potential Distribution of *Philaenus spumarius*, *Neophilaenus campestris* and *N. lineatus* (Hemiptera, Aphrophoridae) in Southeastern Iberian Peninsula

**DOI:** 10.3390/insects14070592

**Published:** 2023-06-30

**Authors:** Diego Gallego, Sandra Carol Sabah, José Luísis Lencina, Antonio Félix Carrillo

**Affiliations:** 1 Sanidad Agrícola Econex S.L., 30149 Murcia, Spain; sandra.sabah@e-econex.com (S.C.S.); joseluis.lencina@e-econex.com (J.L.L.); 2 Department of Ecology, University of Alicante, 03690 Alicante, Spain; 3 Latizal S.L., 30100 Murcia, Spain; felix@latizal.com; 4 Department of Environmental Sciences and Natural Resources, University of Alicante, 03690 Alicante, Spain

**Keywords:** Auchenorrhyncha, *Xylella fastidiosa* vectors, MaxEnt, habitat suitability

## Abstract

**Simple Summary:**

*Xylella fastidiosa* (Bacteria, Xanthomonadaceae), the grapevine Pierce’s disease agent, is an American native bacterium, considered among the more dangerous invasive pathogens for grape, olive and stone fruit trees in Europe and Asia. *Xylella fastidiosa* is vectorized by sapfeeding insects, such as the native Eurasian spittlebugs *Philaenus spumarius* and *Neophilaenus campestris.* Knowledge of the distribution and habitat preferences of *X. fastidiosa* vectors and other potential vectors is an essential element of contingency plans against future *X. fastidiosa* invasions. Our work presents the results of extensive sampling of *P. spumarius*, *N. campestris*, *N. lienatus* and *Lepyronia coleoptrata* in the Autonomous Community of Murcia (SE Spain) in 2020. We performed habitat suitability models of these species using bioclimatic, landscape and topographical variables. Distributions of *P. spumarius*, *N. campestris* and *N. lineatus* in the Murcia region are mainly driven by bioclimatic and landscape composition variables. The more suitable habitats are in the coldest and wettest areas with a high proportion of forests, possibly related to the summer migratory movements. Instead, all three species are absent from large areas of the region, including the southern third, where models predict no suitability. The results could be a useful tool for contingency planning against a possible *X. fastidiosa* invasion.

**Abstract:**

*Philaenus spumarius* and *Neophilaenus campestris* are the main vectors of the invasive bacteria *Xylella fastidiosa* and key threats to European plant health. Previous studies of the potential distribution of *P. spumarius* reveal that climatic factors are the main drivers of its distribution on the Mediterranean Basin scale. Other local studies reveal that the landscape could also have a role in the distribution of both species of *P. spumarius* and *N. campestris*. Our work is aimed at understanding the role and importance of bioclimatic and landscape environmental factors in the distributions of the vector and potential vector species *P. spumarius*, *N. campestris*, *N. lineatus* and *L. coleoptrata* on a regional scale across the Autonomous Community of Murcia (SE Spain), a region with relevant environmental gradients of thermality and crop intensity. We used sweeping nets for sampling 100 points during eight months in 2020. Using bioclimatic landscape composition and topographical variables, we carried out habitat suitability models for each species using the maximum entropy algorithm (MaxEnt). Distribution results for *P. spumarius*, *N. campestris* and *N. lineatus* indicate a gradient in habitat suitability, with the optimum in the coldest and wettest areas in landscapes with a high proportion of forest. All three species are absent from the southern third of the study region, the hottest, driest and most intensively cultivated area. These results are useful and should be considered in contingency plans against possible invasions of *X. fastidiosa* in Mediterranean regions.

## 1. Introduction

The spittlebug includes a group of sapfeeding species of the suborder Auchenorrhyncha (Hemiptera), composed of five families: Aphrophoridae, Cercopidae, Clastopteridae, Epipygidae and Machaerotidae, whereby in the nymphal stage they produce a protective foam cover, like saliva. Their ability to act as a vector of viruses, phytoplasmas and bacteria, such as the invasive pathogen *Xylella fastidiosa* (Wells), puts representatives of this suborder in a category of important crop pests worldwide. Three species of Aphrophoridae are recognized vectors of *X. fastidiosa* in Europe: *Philaenus spumarius* (Linnaeus, 1758) [1,2], *Neophilaenus campestris* (Fallen, 1805) and *P. italosignus* (Drosopoulos and Remane, 2000) [3]. No other species of Auchenorrhyncha, such as other *Neophilaenus* species, *Lepyronia coleoptrata* (Linnaeus, 1758) or *Cicadella viridis* (Linnaeus, 1758), have been confirmed as vectors in Europe [4].

*Xilella fastidiosa* is the most important pathogen for olive and stone fruit crops in Europe and Asia [5]. The bacterium was included in the A2 of EPPO list as a quarantine pathogen, which was detected in Italy (2013) [6], Spain (2016) [7], Israel (2017–2018) [8] and Iran (2014) [9], evidence of a wide invasive process in the Mediterranean Basin and Asia. Since its first detection in Spain in 2016, in Majorca, *X. fastidiosa* has been detected in most parts of all of the Balearic Islands (except Formentera), infesting more than 30 vegetal species. In 2017, almond trees infected with the bacterium were detected in mainland Spain, in Alicante province (Eastern Iberian Peninsula). This outbreak currently covers an area of 4.611 ha, infecting 21 vegetal species [10].

The way that the climatic drivers determine the distribution of *P. spumarius* has been studied in the Mediterranean Basin by Godefroid et al. [11], predicting high climatic suitability in a great part of Western Europe but moderate or little suitability for the warmest and driest regions of the Iberian Peninsula, eastern Greece and central Turkey. On the other hand, the works of Cornara et al. [2] and Lago et al. [12] indicate that *P. spumarius* and *N. campestris* are species with high mobility that allow migrations to seek adequate plant resources as a surviving strategy during the Mediterranean summer dryness. This denotes the importance of the landscape composition in the habitat preferences of these species. Santoiemma et al. [13] concluded a preference of *P. spumarius* for agroecosystems located in a landscape matrix of olive crops, but with the presence of other crops and grassland. Lago et al. [12] reported that *N. campestris* migrates from olive crops to pine forests in summer. Therefore, both species’ behavior could require living in agro-forest mosaics or non-homogeneous landscapes. In this line, Chartois et al. [14] conclude that factors of different scales drive the abundance of *P. spumarius* in mesomediterranean habitats of Corsica (France): from abiotic (pluviometry and temperature) to biotic factors (abundance of the host plant *Cistus monspeliensis*). 

Our work is aimed at understanding which environmental factors on the regional scale, mainly among bioclimatic and landscape factors, drive the habitat suitability and distribution of the vector species *P. spumarius* and *N. campestris*, and other putative vectors such as *N. lineatus* and *L. coleoptrata*, on a regional scale. Our results could be a useful tool for focusing regional management strategy against the future invasion of *X. fastidiosa* in southeastern Spain areas with a high vector presence, instead of areas where they are absent.

## 2. Materials and Methods

### 2.1. Study Area

**Figure 1:**
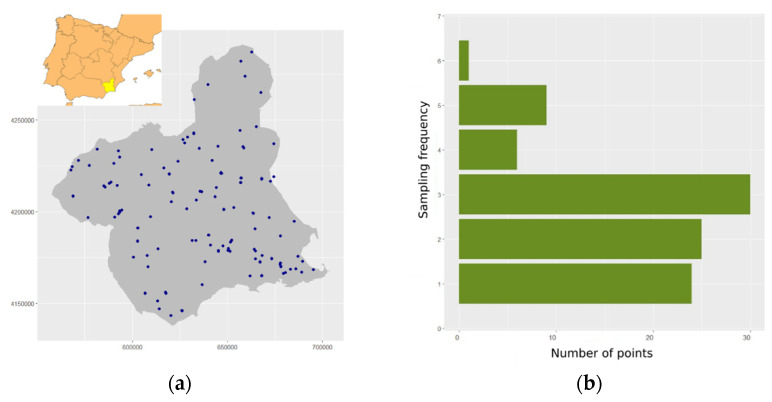
Location of the study area, the Autonomous Community of Murcia Region, and distribution of the sampling points (**a**). Histogram of the sampling frequency of points from May to December 2020 (**b**).

The study area comprises the 11,314 km^2^ of the Autonomous Community of Murcia. This area is located in a dry Mediterranean climate in the southeastern Iberian Peninsula (Figure 1), with 338 mm of annual rainfall and 18 °C of mean annual temperature. The altitude range, from 0 to 2027 m, and the NW–SE dryness gradient define this region as a transition area between the Mediterranean and the subtropical shrub lands, the distribution limit of several vegetal and animal species [15].

### 2.2. Sampling Design

To survey the complete study area and cover the whole flight period of the species, we designed a network of 100 random sampling points. We used vector layers of tree crops and natural or semi-natural vegetation of the Murcia region (downloaded for free from https://www.miteco.gob.es/, accessed on 1 November 2022) to select 100 random points located in the natural–tree crop interface along the study area. The random procedure for the selection of the points was performed using the vectorial basic functions of QGIS [16] (Figure 1). Coordinates, altitude and crop of sample points have been included in Table S1.

A schedule of 12 bi-weekly surveys was organized from mid-May to mid-December 2020. In order to optimize the human and economic resources, a subset of 20 points were randomly selected for each of the 12 surveys. As a result of these selections, the points were surveilled with different frequencies (Figure 1). Most of the points were surveilled one, two or three times. A few points were visited four and five times, although a minority were sampled six times.

In each surveillance, we sampled the insect fauna using sweeping nets with a frame of reinforced stainless steel of 45 cm diameter (ENTO SPHINX s.r.o, Pardubice, Czech Republic). The procedure was modified after Morente et al. [17]. We applied an effort of 200 sweeps per point on natural or semi-natural tree crop canopies and ground cover vegetation. Each sample was immediately transferred to a plastic bag with a hermetical closed zip containing a small amount of ethyl acetate to kill the captured insects. Samples were kept refrigerated (4 °C) until the separation of all arthropod specimens and the identification of the spittlebug species. The samples were stored in the personal collections of the authors.

### 2.3. Distribution and Abundance of Species

We used Raster, maptools and ggplot2 [18,19,20] in R [21] to perform distribution maps of specimen abundance and temporal evolution of the catches of each species.

### 2.4. Distribution Modeling

We used three kinds of spatial data: bioclimatic, landscape and topographical, proportion surfaces of tree crops and natural or semi-natural vegetation, presence vegetation geoseries and topography (Table 1). Bioclimatic layers were obtained from the WorldClim dataset (https://www.worldclim.org/, accessed on 1 November 2022). Other bioclimatic datasets such as Chelsa (https://chelsa-climate.org/, accessed on 1 November 2022) were previously tried and the preliminary results seemed redundant with the WorlClim results; consequently, only the WorldClim dataset was used in this work. Raw vector layers of crops, vegetal formations and geoseries were obtained from https://www.miteco.gob.es/ (accessed on 1 November 2022) and processed using QGIS to obtain raster maps of densities of different environmental units. A digital terrain model (DTM) was downloaded from http://centrodedescargas.cnig.es/, accessed on 1 November 2022, for the calculation of slope and ruggedness index using QGIS. All raster maps were calculated to a detail of ~1 km^2^ (30 arc s). 

Collinearity between variables was avoided using cluster analysis included in the Raster package [18] in R [21]. Highly correlated variables (Pearson’s correlation index > 0.7) were not chosen for modeling.

Distribution models of species were created using the *dismo* and *ENMeval* packages in R [22,23], which use the maximum entropy algorithm MaxEnt for modeling. We used the feature classes linear (L), quadratic (Q), categorical (C) and hinge (H) [24,25,26,27,28,29] for evaluating the best-predicting parameters of combinations of regularization multiplier values (RM) (ranging from 0.5 to 4 with an increment of 0.5) and feature class (FC) combinations (“L”, “LQ”, “H”, “LQH” [11,25]). We used the lowest Akaike information criterion corrected for small sample sizes (AICc, [11,26]) to select the optimal settings of the models. Potential artifactual truncation of response curves was avoided because of inadequate background sampling, using the full representation of environments available for each species by including all of the pixels within the delimited study area [27]. Afterward, we selected, as the best models, the combination of RM and FC associated with the highest mean AUC and spatial cross-validation, through four sets, using the get.block function from the ENMeval R package [30]. Five replicates of each model were performed to reduce the uncertainty induced by the cross-validation methods, processing a consensus prediction for each replicate [11].

Additionally, all of the models were post-evaluated using the dataset “Pres-Abs-Spain1” [11], a binary dataset of the presence and absence of these species. “Pres-Abs-Spain1” was obtained in previous systematic surveys of a 20 fixed point network located in the agro–natural interface of the study area in 2019. The predictive power of each consensus prediction was evaluated using this dataset.

## 3. Results

### 3.1. Sampling Results, Temporal Catches and Abundance Distribution

We collected 8581 specimens of Fulguromorpha and Cicadomorpha, and of which, 20.37% corresponds to vector or potential vector species, that is, 666 specimens of *P. spumarius* (7.76%), 338 *N. campestris* (3.93%), 530 *N. lineatus* (6.17%) and 216 *L. coleoptrata* (2.51%) (Table S1). Considering only the points where the species were collected, the proportions of specimens in the total Fulguromorpha and Cicadomorpha community range from 0.56% to 46.2% for *P. spumarius* (in 29 points), 0.44% to 48.3% for *N. campestris* (in 35 points), 0.3% to 66.7% for *N. lineatus* (in 19 points) and 0.64% to 31.9% for *L. coleoptrata* (in 4 points).

**Figure 2:**
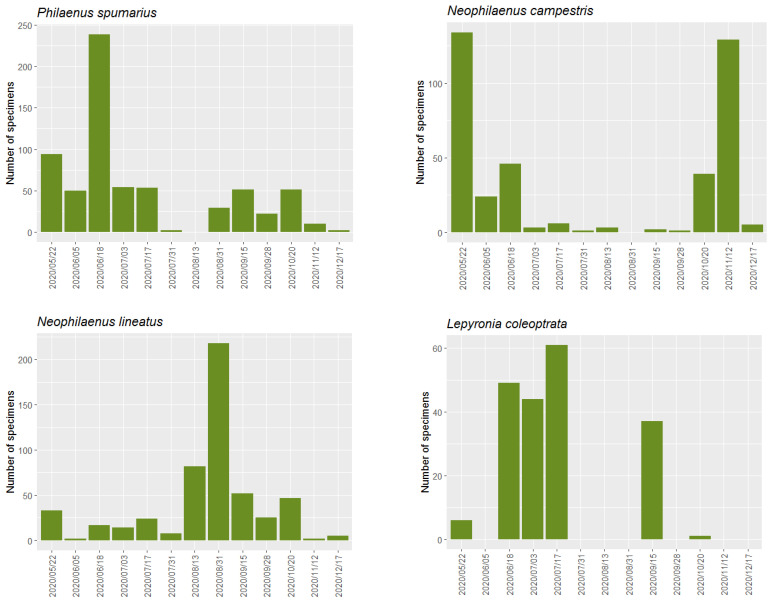
Abundance of *P. spumarius* (up left), *N. campestris* (up right), *N. lineatus* (bottom left) and *L. coleoptrata* (bottom right) in the study area from mid-May to mid-December 2020.

The temporal evolution of catches of these species are shown in Figure 2 and Figure S1. *X. fastidiosa* vectors, *P. spumarius* and *N. campestris* were collected during almost the entire sampling time, from spring to late autumn. It is remarkable to see the lack of captures of both species around mid-summer. The populations of *P. spumarius* show that a clear peak of collections was recorded in mid-June. These capture levels decline from the mid–end of October to mid-December. Instead, *N. campestris* shows two clear peaks of captures, with the first in late spring and the second in mid–late autumn, while during summer and early fall, their populations remain, with a few individuals in the sampled areas.

However, the potential vectors *N. lineatus* and *L. coleoptrata* show different capture patterns. Unlike the vector species, *N. lineatus* was collected in greater numbers from mid-summer to early autumn, with a clear peak in late August. For the rest of the sample period, it was collected in a low number of specimens but somewhat in a greater quantity than its congeneric *N. campestris*. However, *L. coleoptrata* was captured in smaller numbers, mainly from mid-June to mid-July and in mid-September. Null or very few insects were collected from this period.

**Figure 3:**
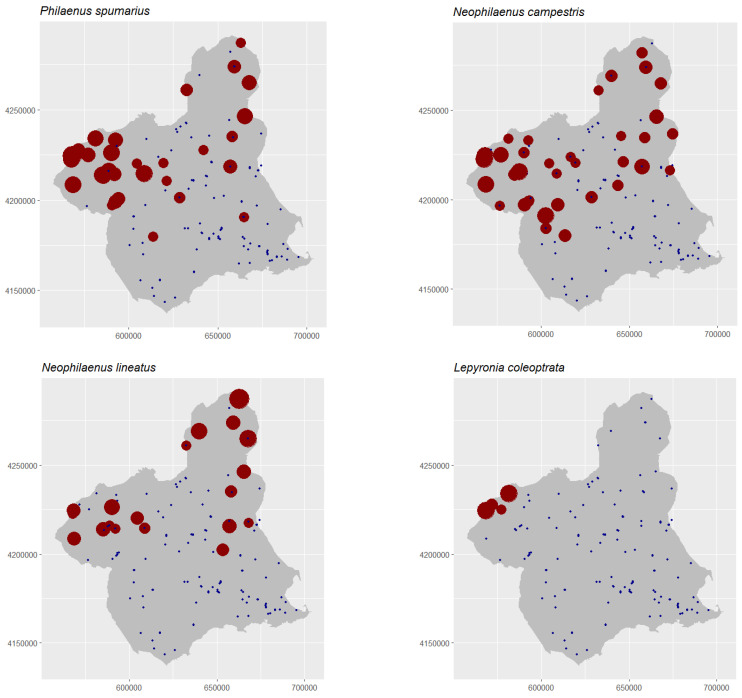
Distribution of collected specimens of *P. spumarius* (up left), *N. campestris* (up right), *N. lineatus* (bottom left) and *L. coleoptrata* (bottom right) in the study area. The size of the red dots is correlated with the number of specimens in Table S1. Absences were plotted in blue. The overlapping of blue and red points indicates that the species was not caught in all surveys of the point.

The spatial distribution of the captures of the four species shows that they were not uniformly distributed throughout the study area (Figure 3). In all of the maps, an area of absence of the species appears to the south or southeast of the study area, except for *L. coleoptrata*, which was exclusively restricted to the northwestern area. The vector species *P. spumarius* and *N. campestris* show similar distributions, although with some differences (Figure 3, upper maps). Both were collected mainly in the northwest and north. *N. lineatus* and *L. coleoptrata* were collected in more restricted areas (Figure 3, bottom maps). The first species, *N. lineatus*, was present in the northwestern and northeastern parts of the study area, separated by an area without collections. The second one, *L. coleoptrata*, was collected exclusively in a restricted northeastern area.

### 3.2. Models of Habitat Suitability

We obtained models of habitat suitability of *P. spumarius*, *N. campestris* and *N. lineatus* species that incorporated more than one kind of variable. No accurate model was obtained for *L. coleoptrata* due to insufficient occurrences in a restricted area.

**Figure 4:**
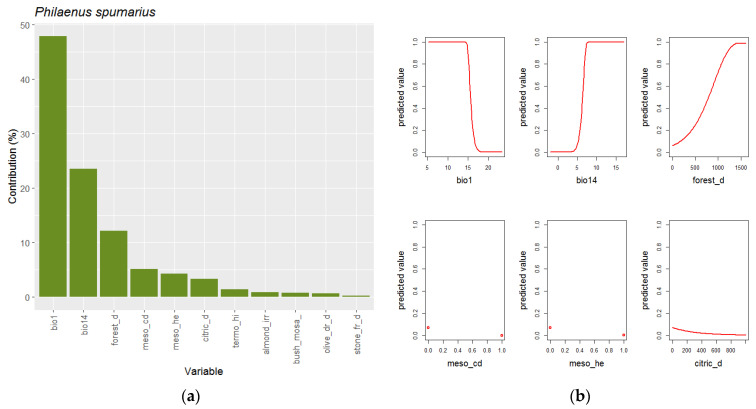
Explanatory variables (**a**) and response curves (**b**) of best models selected by ENMeval for *P. spumarius*. Small red circles indicate responses to binary variables of vegetation geoseries.

The best model of *P. spumarius* (Table 2) incorporates eleven environmental variables, with two bioclimatic variables. The annual mean temperature (Bio1) and precipitation of driest month (Bio14) cumulate together 72% of contribution to the model (Figure 4). The response curves show that *P. spumarius* occurs when the annual mean temperature is less than 15 °C with a transitional range between 15 and 16.5 °C, and the precipitation in the driest month exceeds 8 mm, also with a transitional area between 5 and 8 mm. The other 28% of the model is contributed to by nine landscape and vegetation geoseries variables, and of which, density of forests (forest_d) provides the main contribution (12%). The response curve obtained for this variable indicates that *S. spumarius* is rare in landscapes without a forest and more common when the forest proportion increases (Figure 4). The sum of the other eight variables contributes with less than 16%; so, their response curves are less informative. Nevertheless, the negative relation with both presence of cold and warm mesomediterranean (meso_cd and meso_he) vegetation geoseries and density of citric tree crops (citric_d) is remarkable.

**Figure 5:**
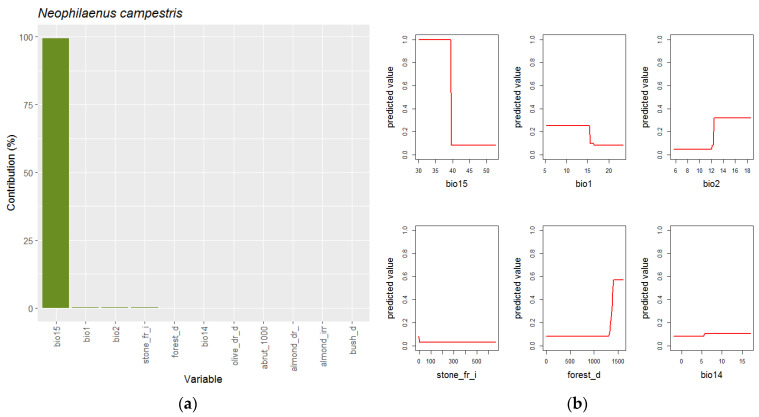
Explanatory variables (**a**) and response curves (**b**) of best models selected by ENMeval for *N. campestris*.

The best model for *N. campestris* also incorporates eleven variables, but only one variable, precipitation seasonality (Bio15), explains the 99.5% (Figure 5). The rest of the marginal explanative variables are all included in bioclimatic or landscape kinds. The Bio15 response curve indicates that the species are mainly present when the coefficient precipitation variation is less than 40, while if the variable exceeds this threshold, *N. campestris* is closely absent. The rest of the variables describe preferences for areas with a mean annual temperature (Bio1) less than 15 °C, mean diurnal range of temperatures more than 13 °C, null presence of irrigated stone fruit crops and high density of forests.

**Figure 6:**
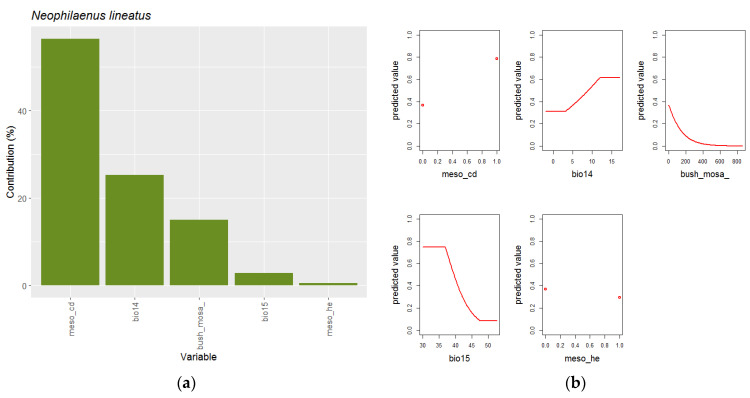
Explanatory variables (**a**) and response curves (**b**) of best models selected by ENMeval for *N. lineatus*. Small red circles indicate responses to binary variables of vegetation geoseries.

The distribution of *N. lineatus* is explained by a model incorporating only five variables (Figure 6). The most explicative variable is the presence of cold mesomediterranean vegetation geoseries (56%), followed by precipitation of driest month (Bio14, 25%), Mediterranean shrub mosaic (Bush_mosa, 15%) and, more marginally, precipitation seasonality (Bio15, 3%) and presence of heat mesomediterranean vegetation geoseries (1%). *N. lineatus* is mainly distributed in cold mesomediterranean areas, with precipitation of the driest month of more than 5 mm and low proportion shrub mosaics.

**Figure 7:**
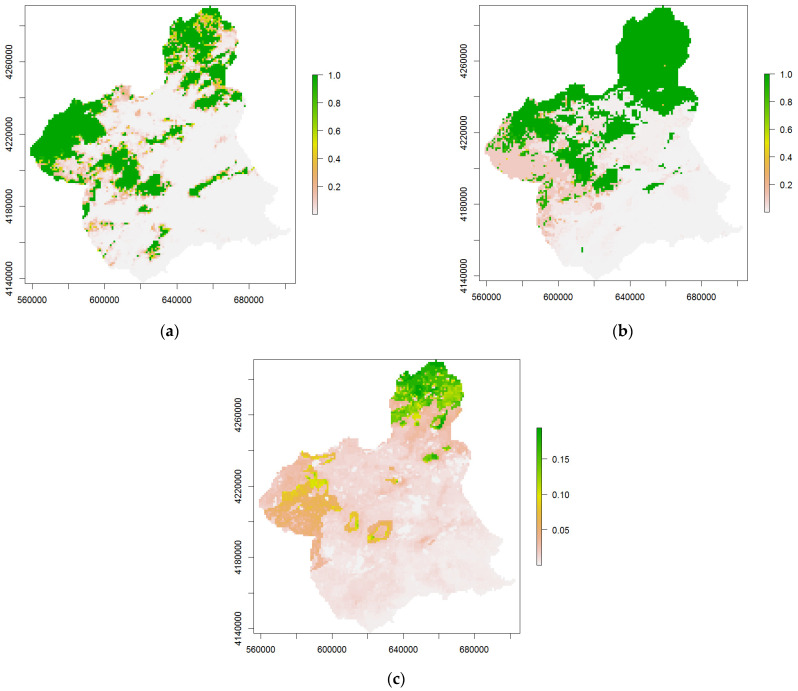
Map of habitat suitability for *P. spumarius* (**a**), *N. campestris* (**b**) and *N. lineatus* (**c**). Scale color bars are MaxEnt logistic outputs, ranging from green (higher suitability) to white (null suitability). Coordinates are in UTM (ETRS89-30N).

The spatial expressions of the habitat suitability models, as potential distribution maps, are shown in (Figure 7). The suitability distribution of *P. spumarius* is shaped by fragmented areas separated by areas of non-suitable habitats (Figure 7a). The biggest continuous area of suitable presence is in the northwestern part of the study area, more or less connected with other suitable habitats in the southeastern part. Another wider suitable area is seen in the north of the Murcia region, but it is less continuous.

The spatial expression of the *N. campestris* model (Figure 7b) predicts a continuous, suitable area in the north as well as other more fragmented suitable areas in the center of the study area, being less suitable to the west and no longer suitable in the southeast. However, the prediction map obtained for *N. lineatus* (Figure 7c) achieves low accuracy, only reaching a maximum suitability index lower than 0.2, mainly predicting the maximal suitability habitat in the north of the study area and diffusing the suitability in the eastern part of the region.

## 4. Discussion

All four species are relatively common in northern and northwestern areas and turn rare to absent toward the southern study area. Considering all of the capture data from all of the samples, the number of individuals of these four species is close to a quarter of the total number of captures. The abundance of the vectors *P. spumarius* and *N. campestris* can range from half of the population specimens in the most abundant sites (46.2 and 48.3 %, respectively) to very scarce in the marginal distribution sites (0.56 and 0.44%, respectively). These values are lower for *P. spumarius* but higher for *N. campestris* than those reported by Bodino et al. [31]. These authors report 79.4% of *P. spumarius* and 20.6% of *N. campestris* for populations in Apulia (northwestern Italy) and 73.8% and 14.7% for both species, respectively, in Liguria (southeastern Italy).

The progressive rarefication of these species toward southeastern areas could be because this geographical zone constitutes the end of the distribution of the four species. Beal et al. [32] reported a very low average number of *P. spumarius* specimens in California vineyards (max. 17.10 individuals/plot), with a sampling effort of 75 sweeps/plot, i.e., 0.228 insects per sweep. This value is close to our maximum mean yield of 0.247 *P. spumarius*/sweeps per plot (49.5 specimens/200 sweeps per plot). Considering that *P. spumarius* is invasive in North America [32], possibly from populations of the Iberian Peninsula and British Islands [33,34], it is possible that a similar distribution scenario of *P. spumarius* in California vineyards as in southeastern Spain cannot be denied.

All of the species considered in our work are univoltine, although the results of our captures seem to be the opposite due to the two adult capture peaks in *P. spumarius*, *N. campestris* and *L. coleoptrata* in late spring and autumn, with a shortage in summer. Tsagkarakis et al. [35] and Drosopoulos and Asche [36] obtained similar results and proposed a bivoltine cycle for *P. spumarius* in Greece. Morente et al. [17] do not follow this proposal because no egg or nymph evidence of a summer generation was reported. The absence of both species in summer was explained by local migration after the dryness of vegetation: the insects left their hatching areas and flew to habitats with nutritive host plants [21,37]. Our data corroborate the migration hypothesis because no nymph evidence was observed in any sampled points in summer.

*Neophilaenus campestris* has the same pattern of summer migration behavior as *P. spumarius*, as Bodino et al. [31] reported and Beal et al. [32] corroborated. This behavior is also supported by our data in the study area. This migration was not observed for *N. lineatus*, which shows a clear peak in mid-summer, evidencing that the species remains in the agro–natural interface during the summer dryness. Unlike congeneric species, this behavior may be related to life strategies to reduce interspecific competition, an issue that must be resolved in further work.

The hypothesis of distribution limit is also sustained by the results of the spatial distribution of captures, showing wide areas where the four species are absent. The main parts of the distributions of *P. spumarius* and *N. campestris* overlap, and both species are absent from the southern third of the study area. The occurrence pattern of *N. lineatus* seems to be more restricted. Both species were captured in two separate areas, in the northwestern and northeastern parts of the studied region. The most restricted distribution corresponds to *L. coleoptrata*, captured only in four points located in the northwestern end of the region. So, it is very plausible that the distribution limits of these four species cross the southeastern region of the Iberian Peninsula.

The regional distributions of *P. spumarius*, *N. campestris* and *N. lineatus* are explained by the MaxEnt models, but for *L. coleoptrata*, any model explains its distribution because of the low number of occurrence points. The distribution of *P. spumarius* is driven mainly by bioclimatic and landscape composition variables. Thus, the maximal habitat suitability is located in the coldest sites, the wettest sites in summer and landscapes with a high proportion of forest areas. So, the model explains the null suitability of this species in the warmest and driest areas, landscapes without a forest and areas with a relatively high proportion of citric crops. The model explains the absence of *P. spumarius* in wide areas of the studied territory, i.e., in transformed landscapes due to intensive crops. Physiological limitations of *P. spumarius* related to temperature were revised by Cornara et al. [38], reporting limitations of nymphal development between a threshold of 2.8 and 26.7 °C [39]. Obviously, the annual temperatures of our study area should not be a physiological limiting factor. However, on the other hand, Godefroid et al. [11] refer to the fact that the dry and warm lowlands of southern Spain are not suitable habitats for *P. spumarius*. Nevertheless, the authors remark that additional factors such as landscape structure, intensive crops and human disturbances must be important in their distribution. Our results indicate that the species are absent in the warmest and driest areas without forests, where intensive crops are established. Nevertheless, islands of favorable conditions (i.e., mountainous lands) allow for the presence of this species. Following our data, in landscapes with traditional or non-intensive crops, in a forest mosaic matrix, where the summer dryness permits the maintenance of vegetal resource diversity, *P. spumarius* finds its maximal habitat suitability. The relation between ecosystem diversity and summer migrations of this species should be investigated in further research.

The distribution model of *N. campestris* incorporates 11 variables, although it is mainly explained by a single climatic variable: precipitation variability. The other ten variables only contribute residually to the explanation. *Neophilaenus campestris* has narrow preferences for areas with low precipitation variability in the study area. Then, *N. campestris* evidences low specialization for its habitat, only requiring landscapes with enough precipitation stability, possibly related to the maintenance of vegetation refuges for assuring migration during the summer.

The model of *N. lineatus* describes preferences for landscapes in the cold mesomediterranean domain, with relatively high precipitations in summer, in a mosaic with a high proportion of bushes, and with relatively low variation in annual precipitations. However, this description of the landscape is not different from that of the previous species (*P. spumarius* and *N. campestris*) and could not be used to explain why *N. lineatus* does not migrate outside of the cropland field borders during the summer, as our data show. On the other hand, unlike both former species, the model of *N. lineatus* does not show rapid changes, possibly evidencing a greater eurioic strategy. This species has usually been captured near small permanently wet environments, such as irrigation ditch losses or small phreatic waterlogging, that maintain small patches of permanent vegetation. Possibly, the fine distribution of this species is not dependent on the regional climate but on the presence of habitats with permanent plant resources, which act on a more detailed scale than that of the landscape.

Incorporating landscape factors improves the accuracy of the previous model proposed by Godefroid et al. [11] for *P. spumarius* in southeastern Spain. In fact, Godefroid and Duran [40] proposed that the forest composition of the landscape was positively associated with the *P. spumarius* and *N. campestris* presence in southwestern Spain. The mesoscale of landscape variables is possibly more suitable for understanding the distribution on a regional scale. Our models evidence a north–south gradient, mainly driven by the temperature, quantity and stability of precipitation and the proportion of forests, with high to zero habitat suitability, compatible with a distribution limit for *P. spumarius*, *N. campestris* and *N. lineatus* throughout the study area. Thus, approximately, the southern third of the region could be considered to have an absence of three species. These results constitute a useful tool and should be considered in contingency plans, given the possibility of the invasion of *X. fastidiosa* in southeastern Spain and other semi-arid areas, allowing one to focus on areas where the vector species are present.

## Figures and Tables

**Table 1:** Type, name and description of the spatial layer of geospatial data used for modeling species habitat suitability.

Kind of Data/Units	Layer	Description
**Bioclimatic**		
°C, mm	Bio1	Annual mean temperature
	Bio2	Mean diurnal range (mean of monthly (max. temp–min. temp))
	Bio3	Isothermality (BIO2/BIO7) (×100)
	Bio4	Temperature seasonality (standard deviation ×100)
	Bio5	Max. temperature of warmest month
	Bio6	Min. temperature of coldest month
	Bio7	Temperature annual range (BIO5-BIO6)
	Bio8	Mean temperature of wettest quarter
	Bio9	Mean temperature of driest quarter
	Bio10	Mean temperature of warmest quarter
	Bio11	Mean temperature of coldest quarter
	Bio12	Annual precipitation
	Bio13	Precipitation of wettest month
	Bio14	Precipitation of driest month
	Bio15	Precipitation seasonality (coefficient of variation)
	Bio16	Precipitation of wettest quarter
	Bio17	Precipitation of driest quarter
	Bio18	Precipitation of warmest quarter
	Bio19	Precipitation of coldest quarter
**Landscape, tree crops**		
0.06 ha/km^2^	Almond_irr	Density of irrigated almond tree crops
	Almond_dr	Density of rainfed almond tree crops
	Olive_irr	Density of irrigated olive tree crops
	Olive_dr	Density of rainfed olive tree crops
	Vineyard_irr	Density of irrigated vineyards
	Vineyard_dr	Density of rainfed vineyards
	High_vineyard	Density of table grape crops
	Citric_d	Density of citric crops
	Stone_fr_irr	Density of irrigated stone fruit tree crops
	Stone_fr_dr	Density of rainfed stone fruit tree crops
**Landscape, natural vegetation**		
ha/km^2^	Forest_d	Density of forests
	Forest_mos	Density of forest mosaic
	Bush_d	Density of Mediterranean shrubs
	Bush_mos	Density of Mediterranean shrub mosaic
	Pasture	Density of grass formations
	Wveg	Density of areas without vegetation
**Landscape, vegetation geoseries**		
presence/absence (binary)	Oro	Presence of oromediterranean zone
	Supra	Presence of supramediterranean zone
	Meso_cd	Presence of cold mesomediterranean zone
	Meso_he	Presence of warm mesomediterranean zone
	Termo_hi	Presence of upper thermomediterranean zone
**Topography**		
m, degrees	Altitud	Mean altitude
	Abrupt	Mean ruggedness index
	Slope	Mean slope

**Table 2:** Parameter setting of MaxEnt from the results of ENMeval of best models of *P. spumarius*, *N. campestris* and *N. lineatus*; AUC: area under curve; RM: regularization multiplier.

Species	AUCtest	AUCdiff	RM	FC ^1^	Explaining Variables
*P. spumarius*	0.839	0.107	4	L	11
*N. campestris*	0.799	0.1577	0.5	LQH	11
*N. lineatus*	0.853	0.096	4	L	5

^1^ L: linear, Q: quadratic, H: hinge.

## Data Availability

Data is contained within the article or Supplementary Materials.

## Supplementary Materials

The following supporting information can be downloaded at https://www.mdpi.com/article/10.3390/insects14070592/s1: Table S1: Coordinates, altitude, crop and total specimen captures of sample points. Figure S1: Maps of specimen abundance and temporal evolution of catches. Size of red point is correlated with the number of specimens of Table S1.
