# Supplementary material for: Bioclimatic and Landscape Factors drive the Potential Distribution of Philaenus spumarius, Neophilaenus campestris and N. lineatus (Hemiptera, Aphrophoridae) in Southeastern Iberian Peninsula
Source: Insects. 2023 Jun 30;14(7):592. doi: 10.3390/insects14070592 (PMC10380695; doi:10.3390/insects14070592)

Philaenus spumarius
2020/05/22

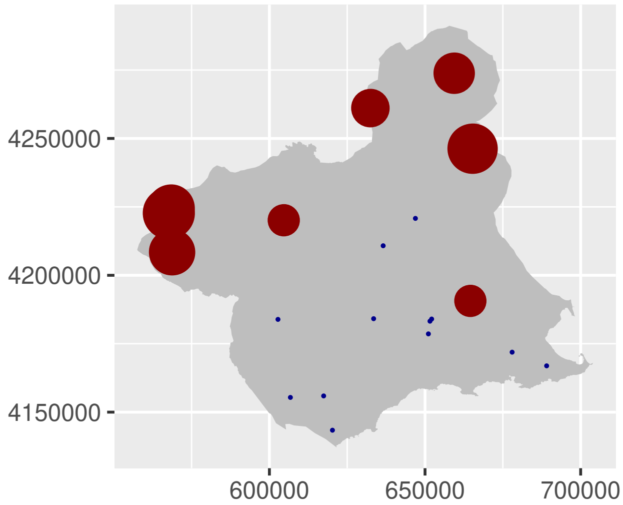

Philaenus spumarius
2020/06/18

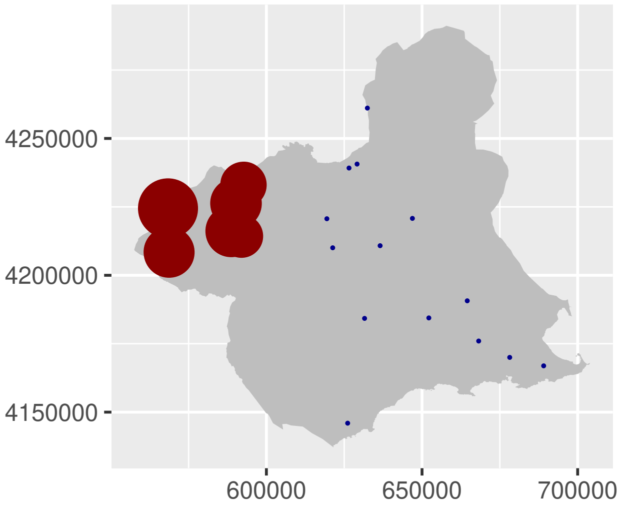

Philaenus spumarius
2020/06/05

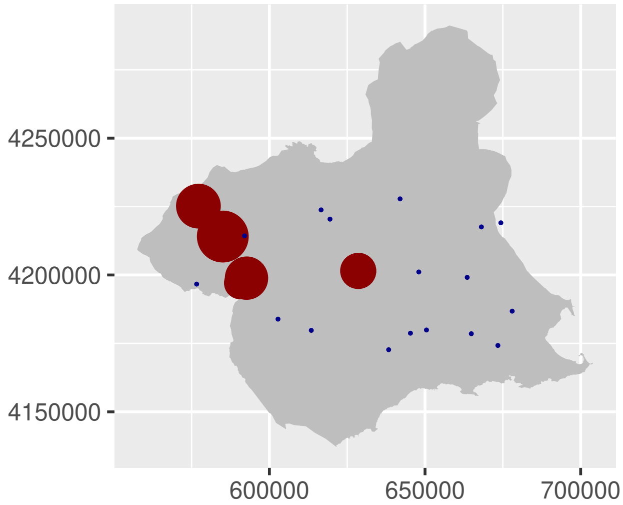

Philaenus spumarius
2020/07/03

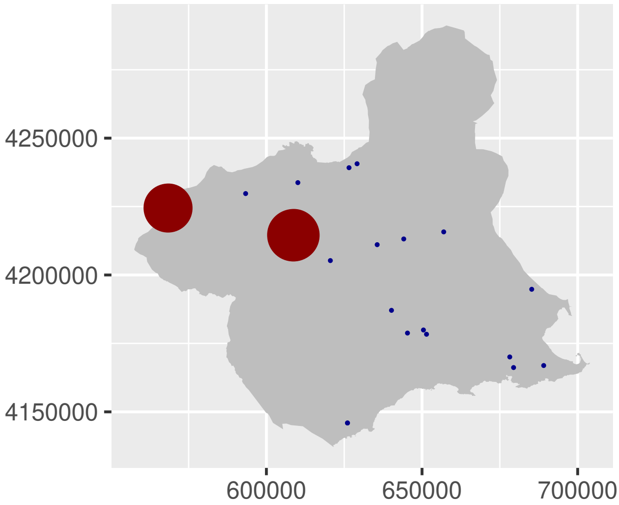

Philaenus spumarius
2020/07/17

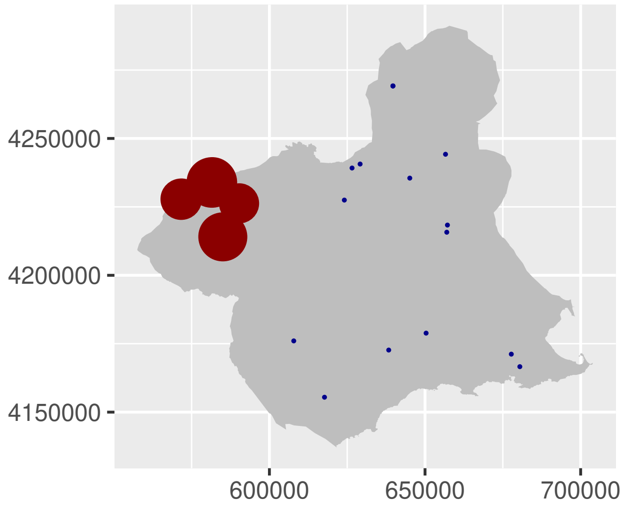

Philaenus spumarius
2020/08/13

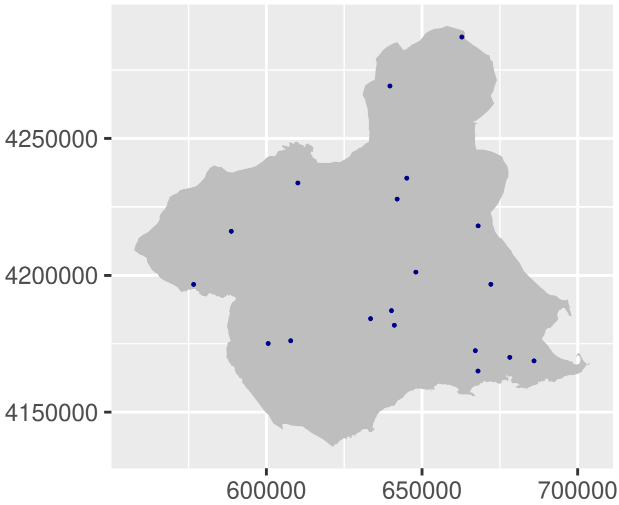

Philaenus spumarius
2020/07/31

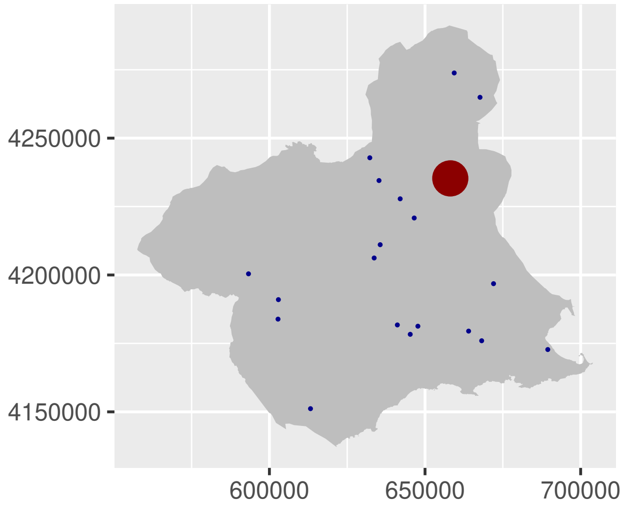

Philaenus spumarius
2020/08/31

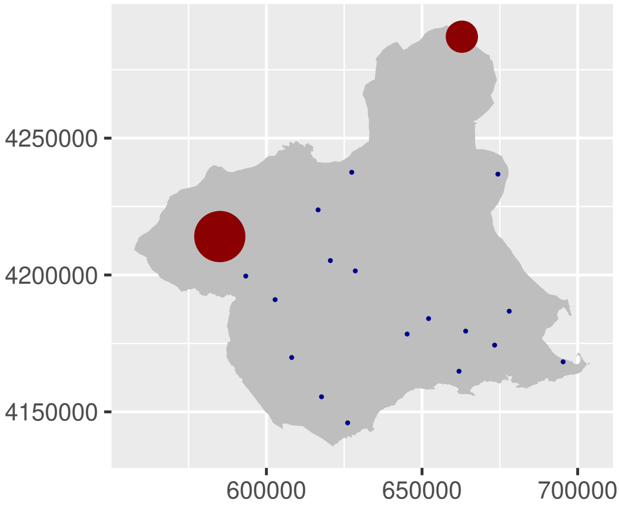

Philaenus spumarius

2020/09/15

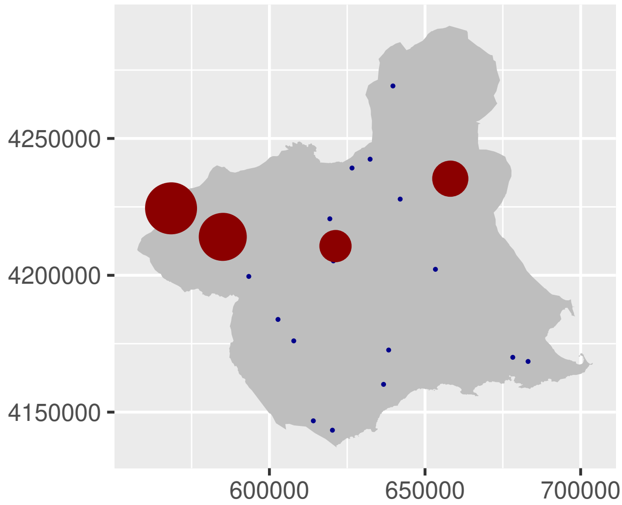

Philaenus spumarius

2020/10/20

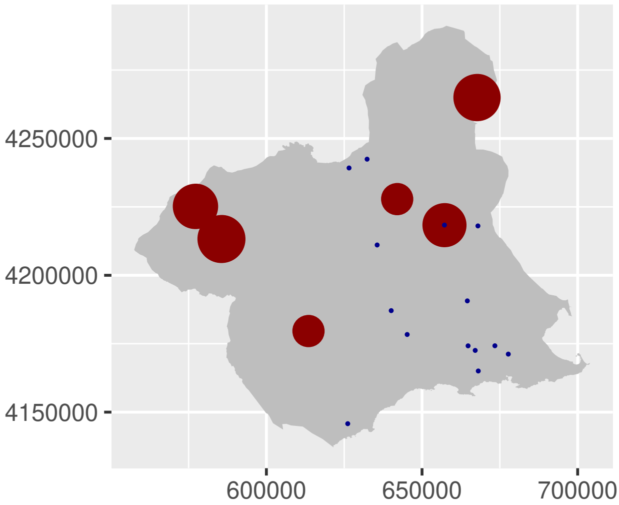

Philaenus spumarius

2020/09/28

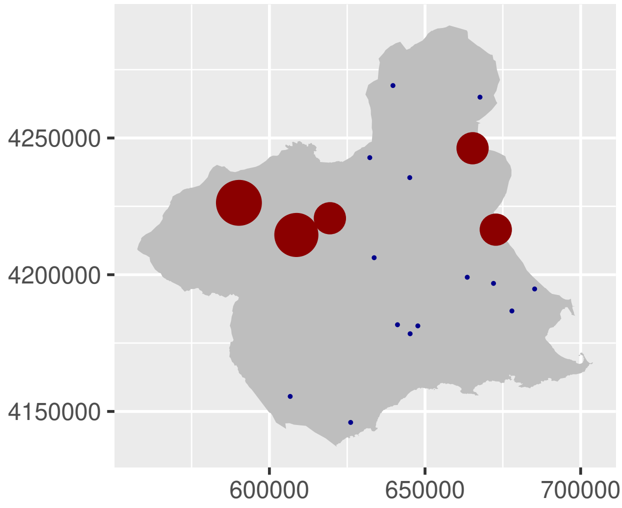

Philaenus spumarius

2020/11/12

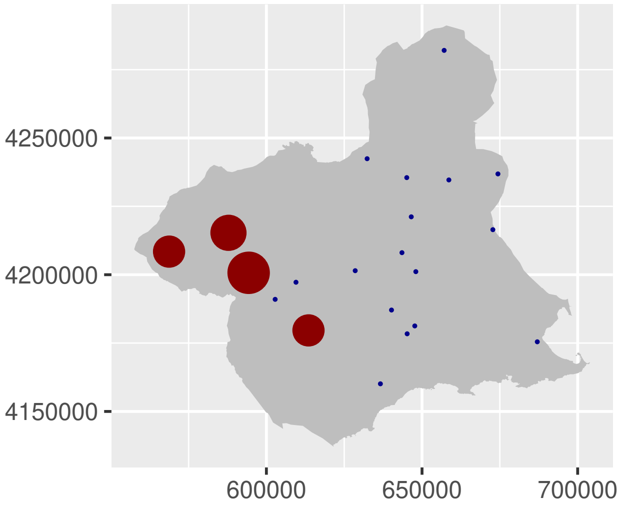

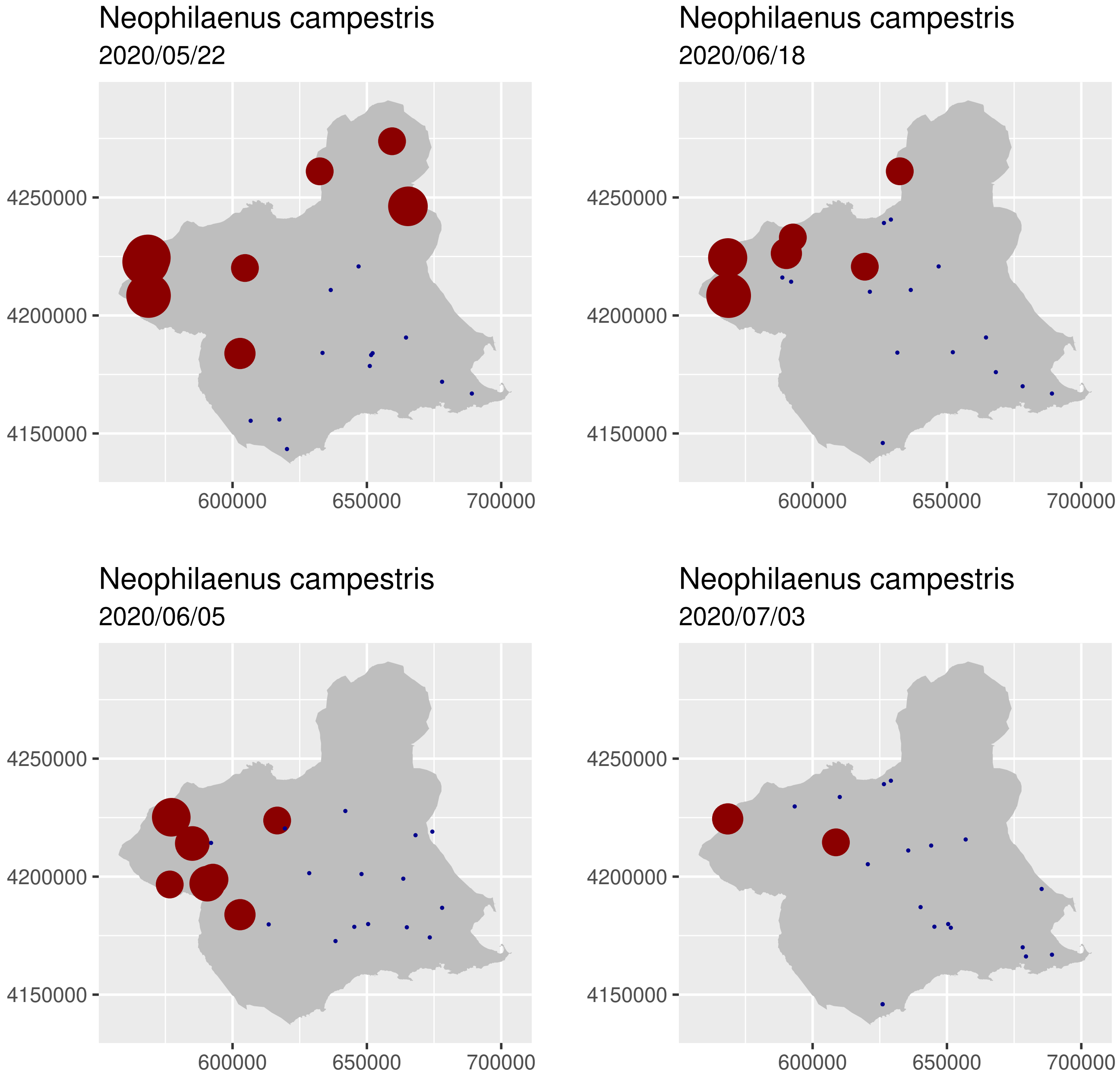

Neophilaenus campestris
2020/05/22
Neophilaenus campestris
2020/06/18
Neophilaenus campestris
2020/06/05
Neophilaenus campestris
2020/07/03
4250000
4200000
4150000
600000
650000
700000

Neophilaenus campestris
2020/07/17

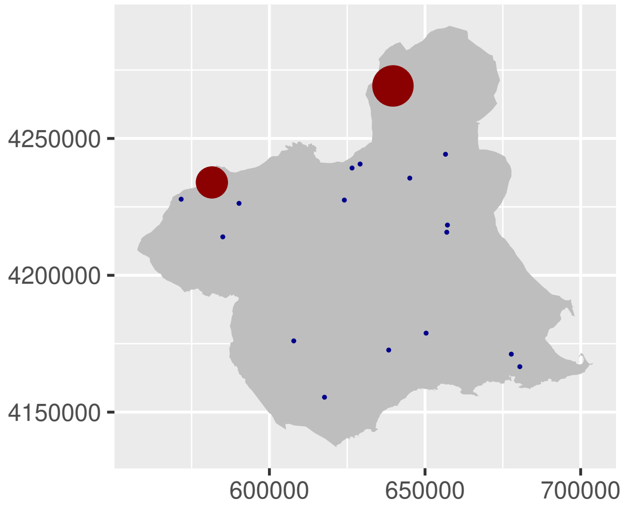

Neophilaenus campestris
2020/08/13

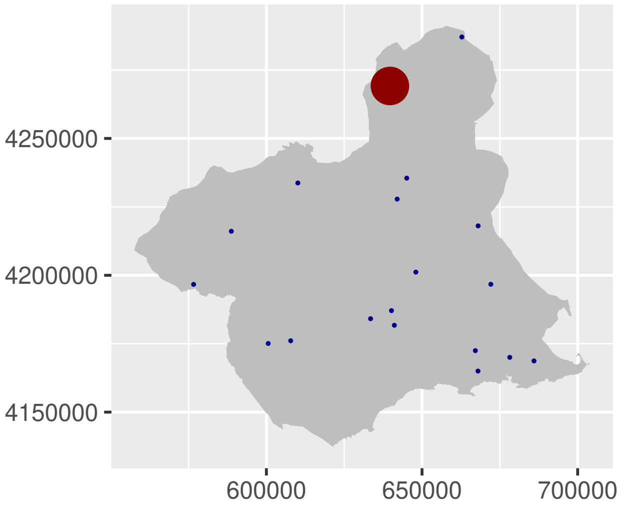

Neophilaenus campestris
2020/07/31

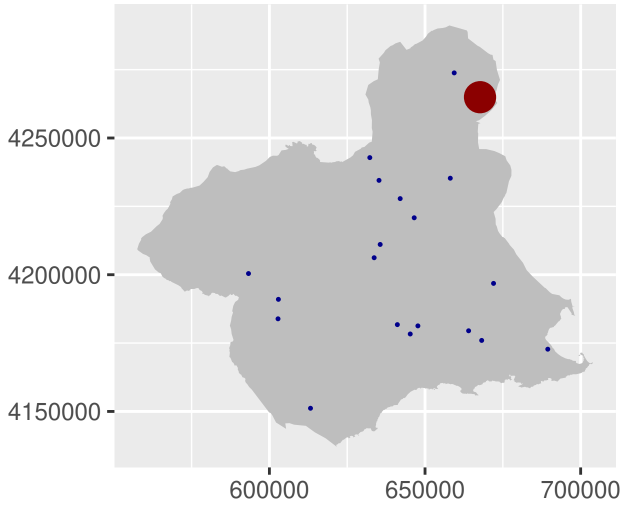

Neophilaenus campestris
2020/08/31

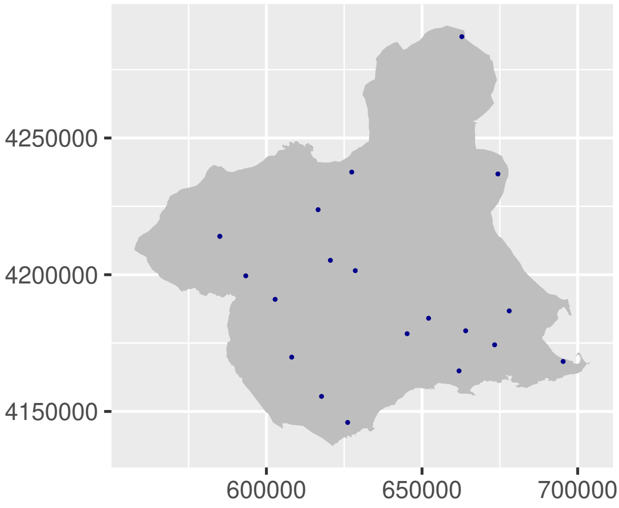

Neophilaenus campestris
2020/09/15

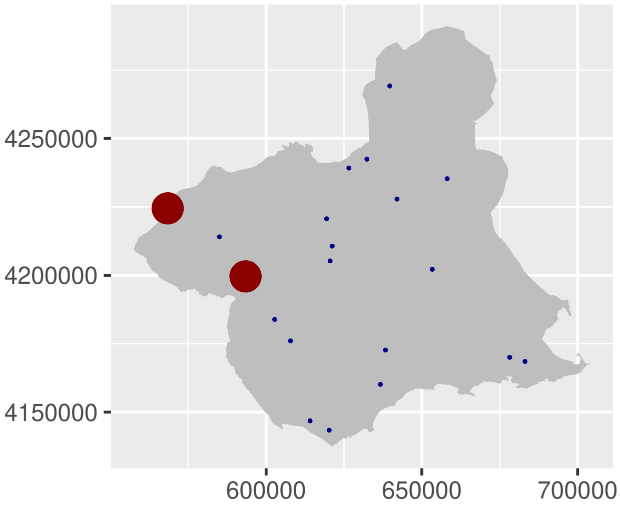

Neophilaenus campestris
2020/10/20

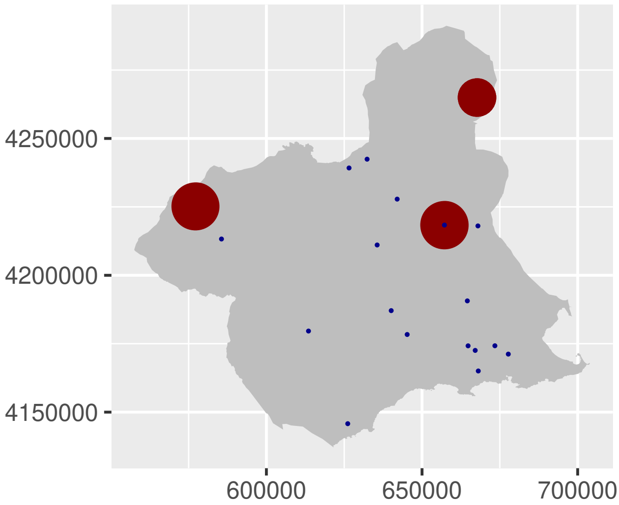

Neophilaenus campestris
2020/09/28

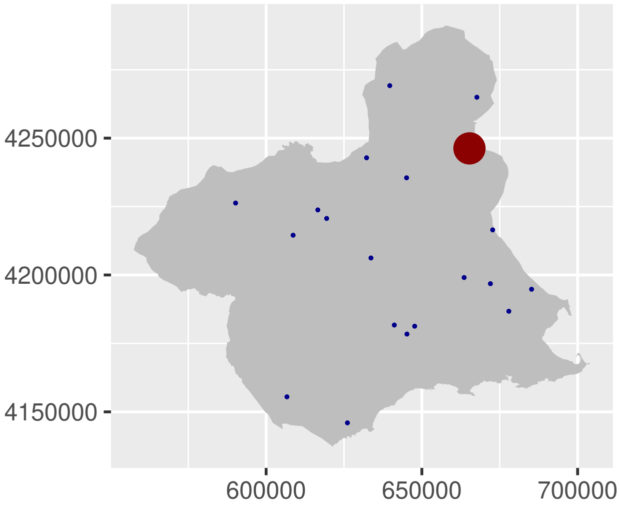

Neophilaenus campestris
2020/11/12

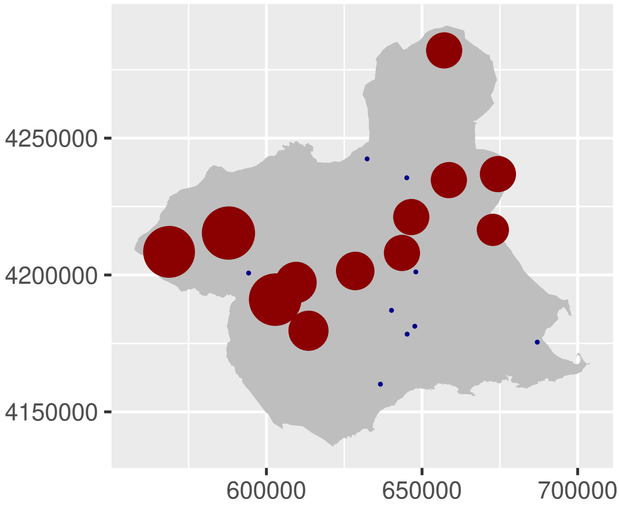

Neophilaenus lineatus
2020/05/22

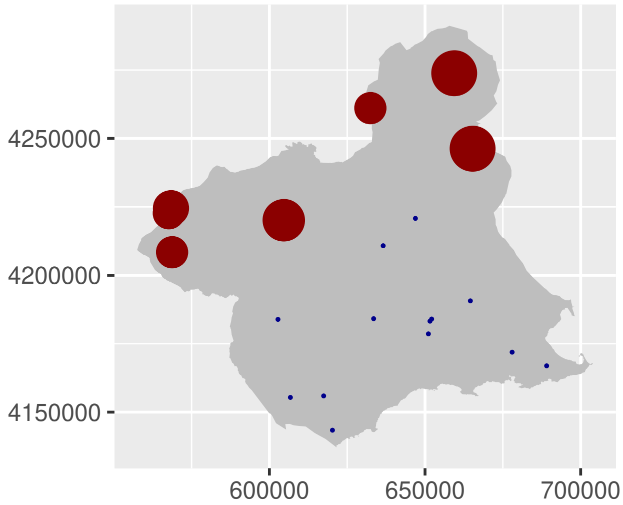

Neophilaenus lineatus
2020/06/18

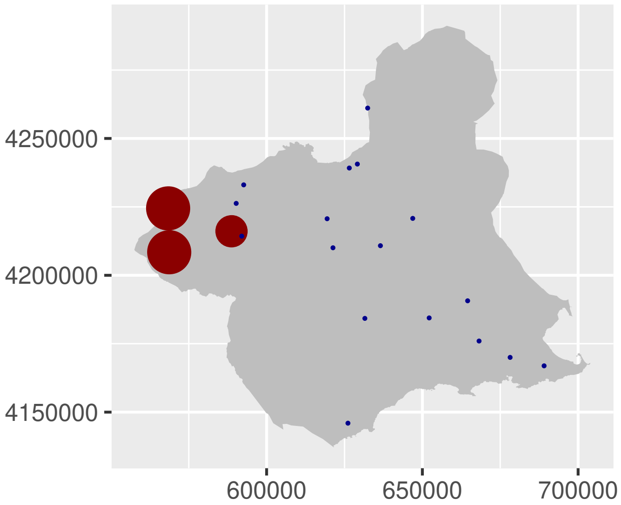

Neophilaenus lineatus
2020/06/05

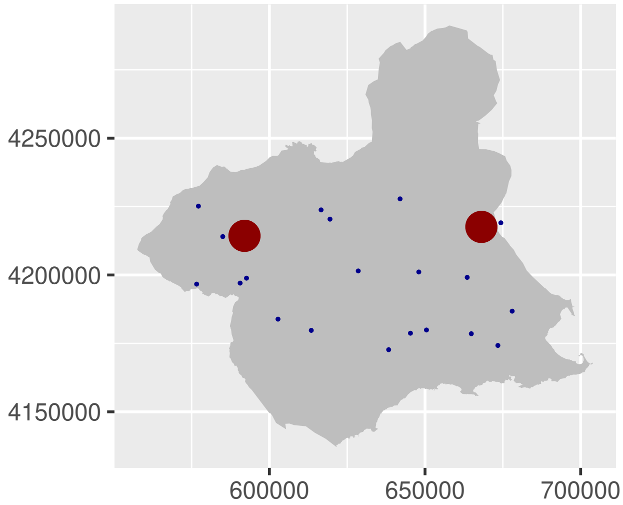

Neophilaenus lineatus
2020/07/03

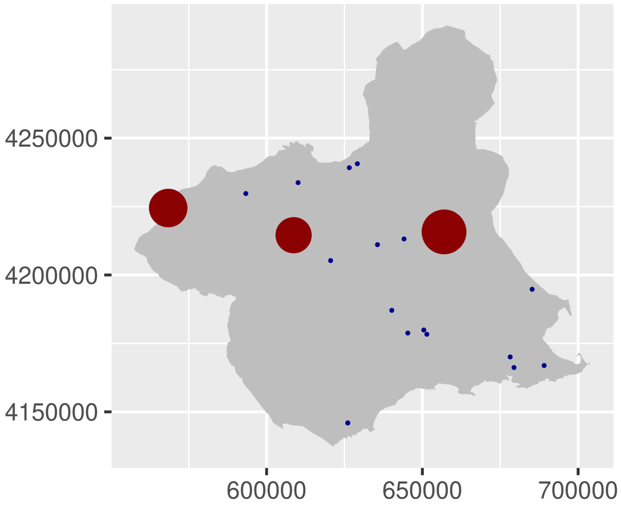

Neophilaenus lineatus
2020/07/17

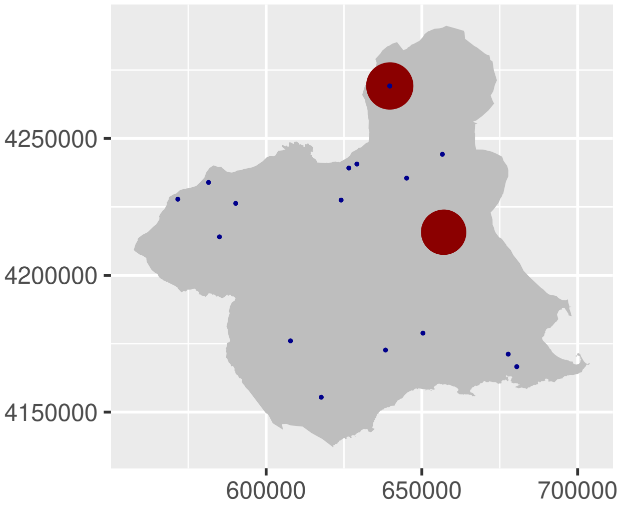

Neophilaenus lineatus
2020/08/13

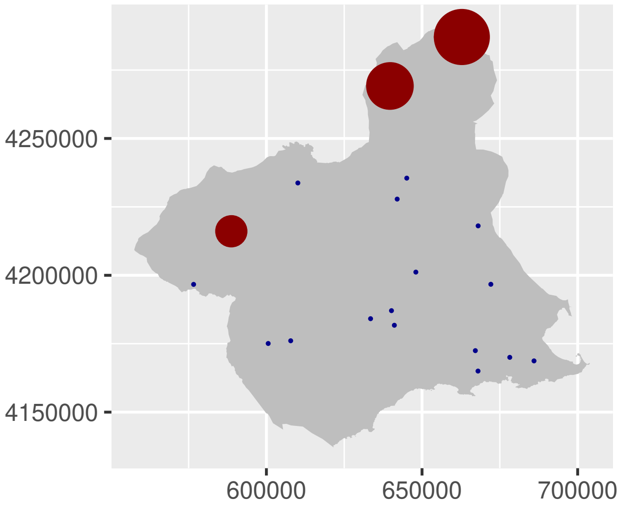

Neophilaenus lineatus
2020/07/31

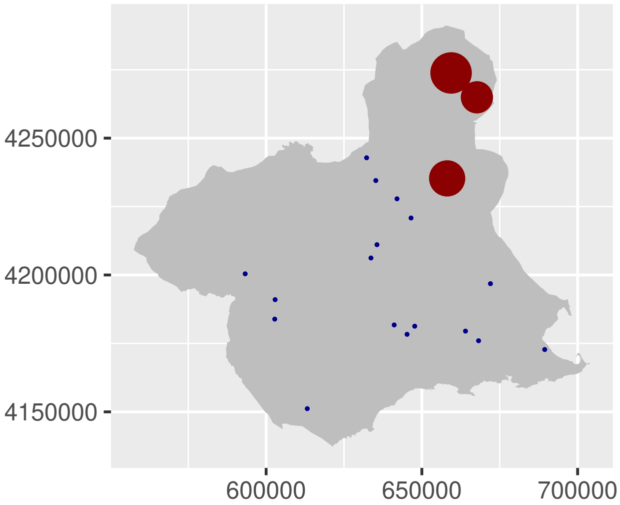

Neophilaenus lineatus
2020/08/31

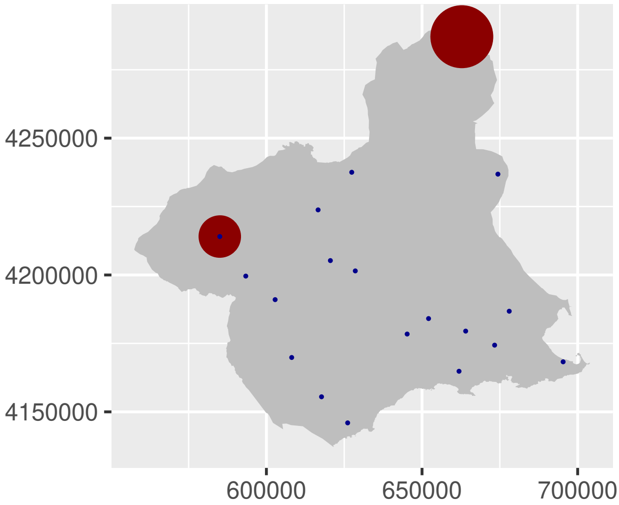

Neophilaenus lineatus

2020/09/15

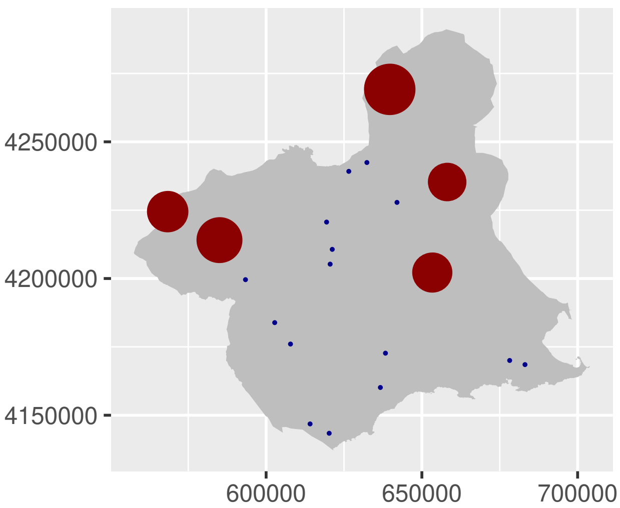

Neophilaenus lineatus

2020/10/20

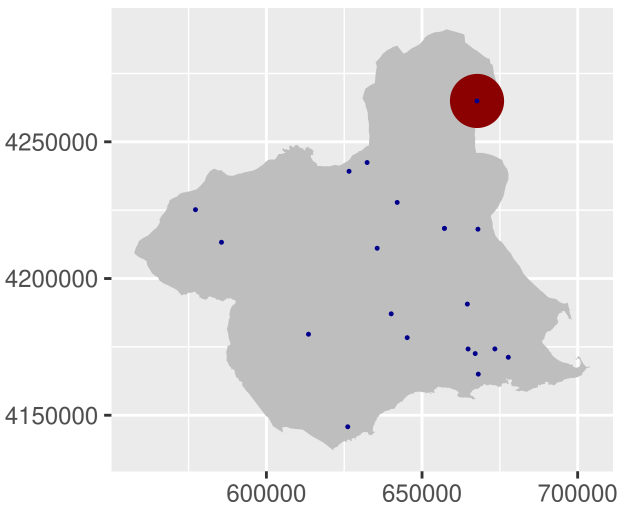

Neophilaenus lineatus

2020/09/28

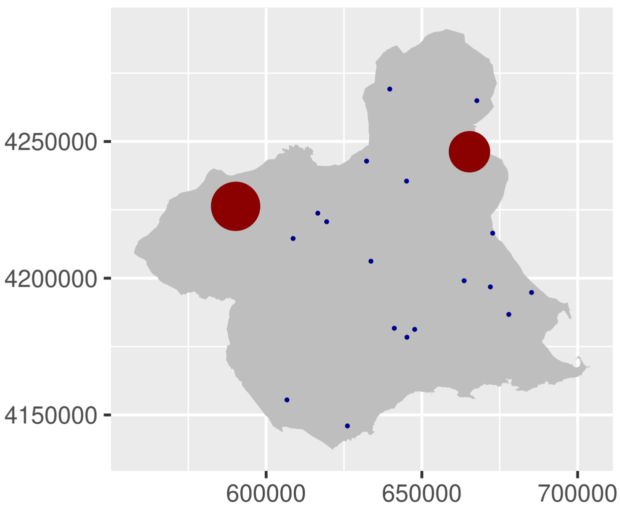

Neophilaenus lineatus

2020/11/12

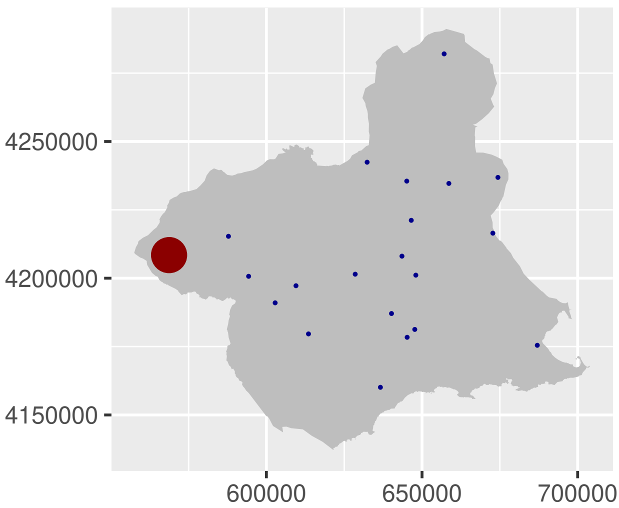

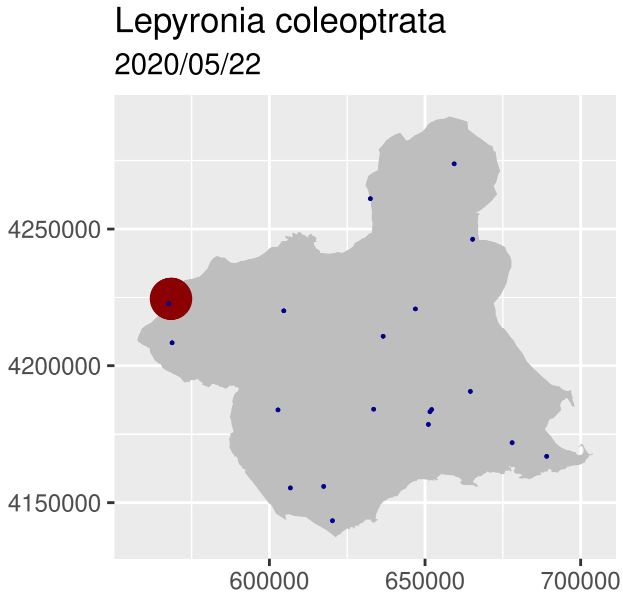
Lepyronia coleoptrata
2020/05/22
4250000
4200000
4150000
600000
650000
700000

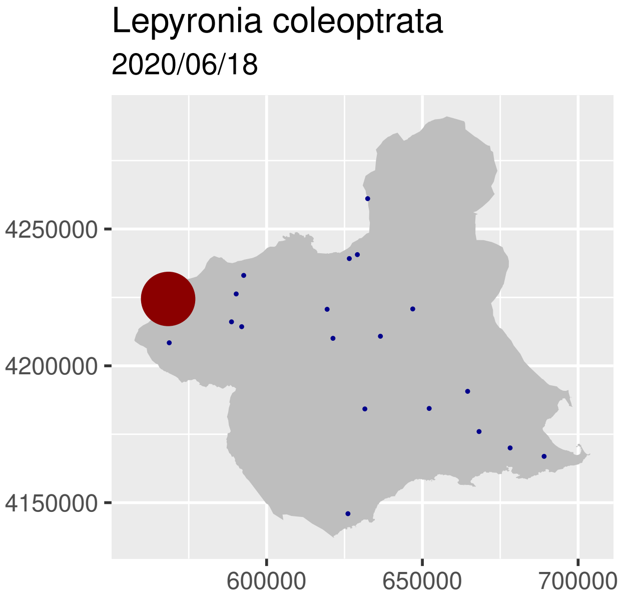
Lepyronia coleoptrata
2020/06/18
4250000
4200000
4150000
600000
650000
700000

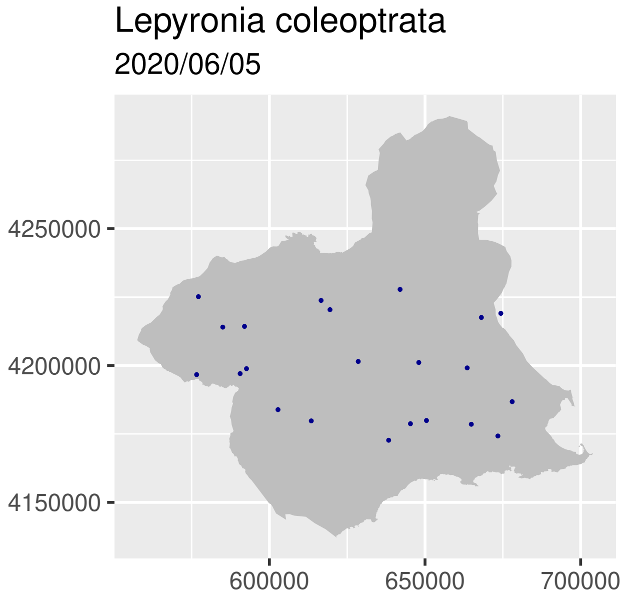
Lepyronia coleoptrata
2020/06/05
4250000
4200000
4150000
600000
650000
700000

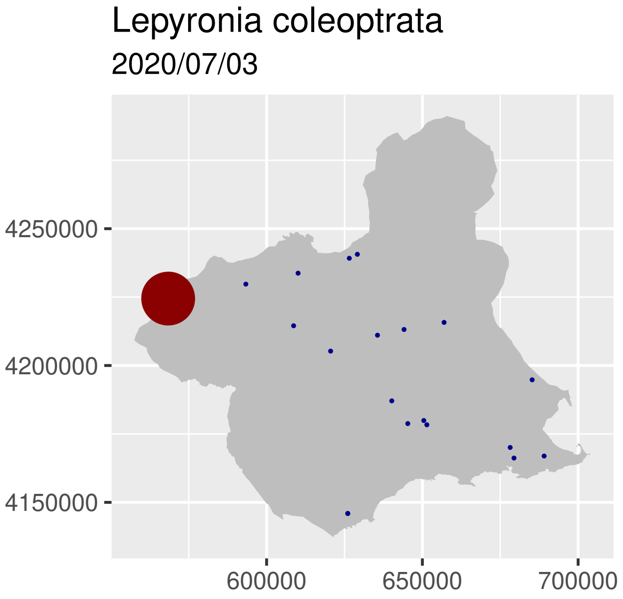
Lepyronia coleoptrata
2020/07/03
4250000
4200000
4150000
600000
650000
700000

Lepyronia coleoptrata
2020/07/17

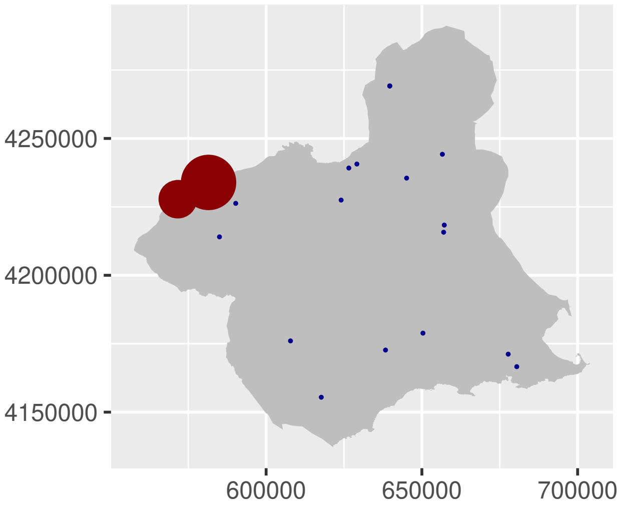

Lepyronia coleoptrata
2020/08/13

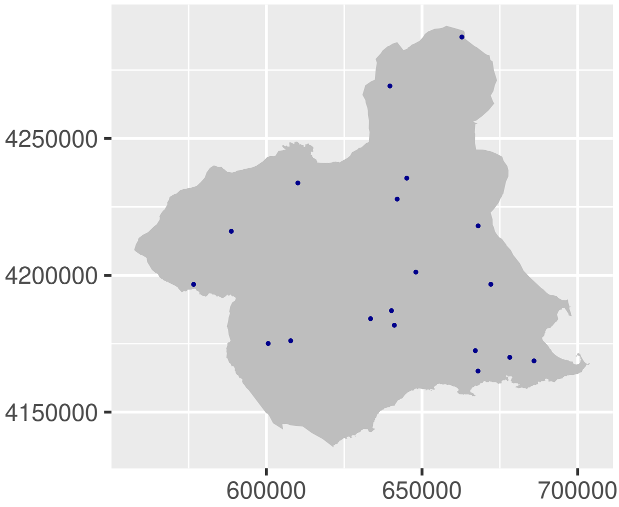

Lepyronia coleoptrata
2020/07/31

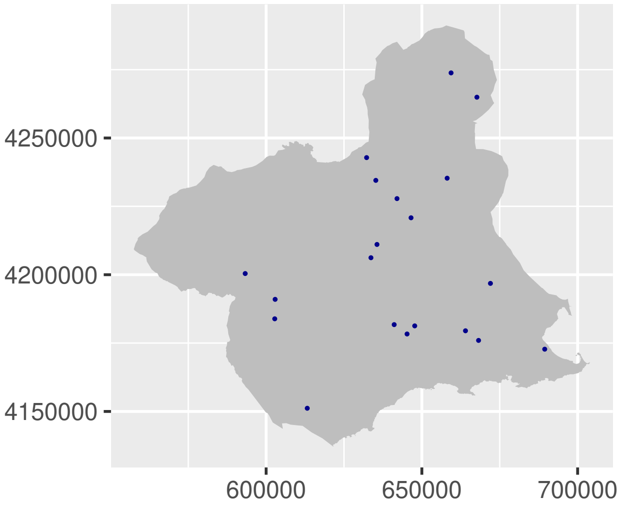

Lepyronia coleoptrata
2020/08/31

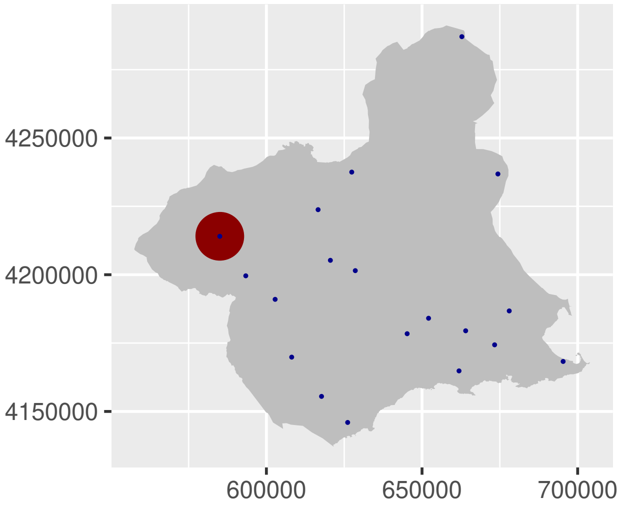

Lepyronia coleoptrata
2020/09/15

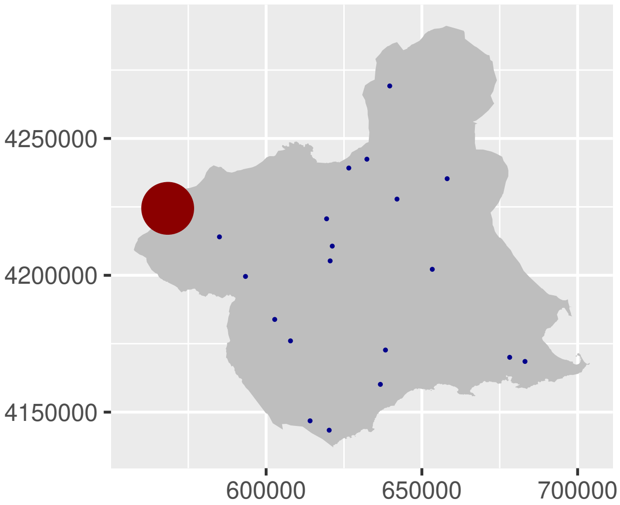

Lepyronia coleoptrata
2020/10/20

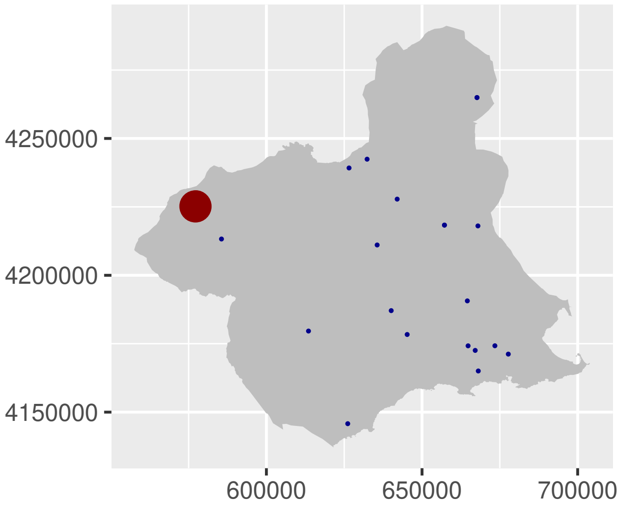

Lepyronia coleoptrata
2020/09/28

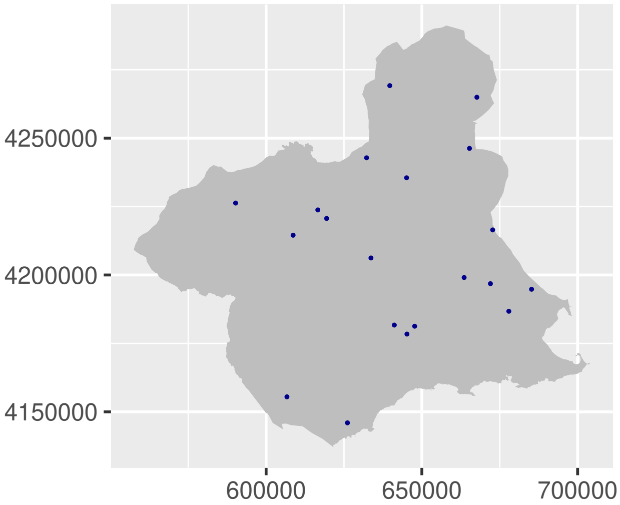

Lepyronia coleoptrata
2020/11/12

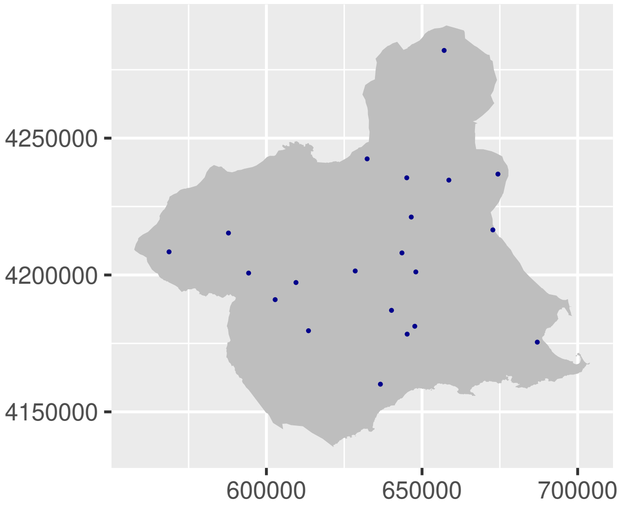

Supplement: Supplementary file 1 [file insects-14-00592-s001.zip › insects-2418588 - Supplementary/Figure S1.pdf]
